# Spotlights on extracellular vesicles in hepatocellular carcinoma diagnosis and treatment: an update review

**DOI:** 10.3389/fbioe.2023.1215518

**Published:** 2023-06-29

**Authors:** Caizheng Wang, Xiaoying Zhang, Jiahui Yu, Jiawen Bu, Xi Gu, Yue Wang, Xudong Zhu, Jie Lin

**Affiliations:** ^1^ Department of General Surgery, Huangyan Hospital, Wenzhou Medical University, Taizhou, Zhejiang, China; ^2^ Department of Ultrasound, Shengjing Hospital of China Medical University, Shenyang, Liaoning, China; ^3^ Department of Oncology, Shengjing Hospital of China Medical University, Shenyang, Liaoning, China; ^4^ Department of General Surgery, Cancer Hospital of China Medical University, Liaoning Cancer Hospital and Institute, Shenyang, Liaoning, China

**Keywords:** hepatocellular carcinoma, extracellular vesicles, tumor development, chemoresistance, biomarkers, nanotherapeutic strategies

## Abstract

Hepatocellular carcinoma (HCC), one of the most prevalent cancers, with a high mortality rate worldwide, seriously impairs patient health. The lack of accurate targets impedes the early screening and diagnosis of HCC and is associated with a poor response to routine therapies. Extracellular vesicles (EVs), comprising exosomes, microvesicles, and apoptotic bodies, are lipid bilayer membrane-derived nanometer-sized vesicles. EVs can be secreted from various cancer cells and release diverse biomolecules, such as DNA, RNA, proteins, metabolites, and lipids, making them a potential source of biomarkers and regulators of the tumor microenvironment. Emerging evidence suggests that EVs are involved in intercellular communication by carrying biological information. These EVs elicit physiological functions and are involved in the oncogenesis of HCC, such as proliferation, invasion, metastasis, and chemoresistance of HCC. EVs have also been considered promising biomarkers and nanotherapeutic targets for HCC. Therefore, this review sheds light on the current understanding of the interactions between EVs and HCC to propose potential biomarkers and nanotherapeutic strategies.

## 1 Introduction

Hepatocellular cancer ranks as the sixth most prevalent cancer globally and the fourth leading cause of cancer-related death. It constitutes approximately 75%–85% of all liver tumors (Siegel et al., 2023). Despite advancements in hepatocellular carcinoma (HCC) treatment approaches, the therapeutic response of patients with intermediate to advanced HCC remains low due to factors such as tumor recurrence, metastasis, and drug resistance, resulting in a dismal prognosis (Wang et al., 2022; Zelli et al., 2022). Since their discovery in 1983 (Harding et al., 1983), extracellular vesicles (EVs) have evolved from being perceived as mere cellular waste disposal systems to a novel mechanism of intercellular communication. Notably, non-coding RNAs contained within EVs play a significant role in conveying intercellular information and profoundly influencing tumor malignancy including in HCC (Fabbiano et al., 2020; Kotani et al., 2021). Ning J et al. have found that miR-12–92 cluster originated from EVs of M2 tumor-associated macrophage significantly contributed to the imbalance of TGF-β1/BMP-7 pathways in HCC cells and promoted the invasion and metastasis of HCC by inhibiting TGFBR2/Smad ubiquitylation regulatory factor 1 (Smurf1)/activin A receptor type 1 (ACVR1) signal pathway (Ning et al., 2021). Besides, Han S *et al.* found that miR-3190 was upregulated in EVs originated from HCC cells which metastasized and colonized in bone tissues. The bone-metastasized HCC-derived EVs which were loaded with miR-3190 can be transferred into orthotopic tumor cells and promote their metastasis by dereasing the expression of AlkB homolog 5 (ALKBH5) (Han et al., 2023). Further elucidation of the biological functions of EVs not only revolutionizes our comprehension of intercellular information exchange and tumor progression regulatory mechanisms, but also offers novel perspectives for cancer diagnosis and treatment, such as identifying tumor diagnostic biomarkers based on EVs and developing innovative tumor nanotherapy approaches (Aharon et al., 2021; Coffin et al., 2021). These advances hold promise for early hepatocellular carcinoma (HCC) detection and improved outcomes in HCC patients unresponsive to conventional treatments. Consequently, this review aims to examine the role of non-coding RNAs present in EVs in HCC progression, explore the potential of EVs as HCC diagnostic biomarkers, and summarize emerging EV-based nanotherapy strategies for HCC, thereby providing valuable insights for clinical diagnosis and treatment of HCC.

## 2 The non-coding RNA present in EVs can regulate the progress of HCC and affect its therapeutic sensitivity

The majority of liver cancer patients are diagnosed at intermediate to advanced stages of the disease. At this advanced stage, liver transplantation is no longer viable. The primary treatment alternatives currently available involve a combination of transhepatic arterial chemoembolization (TACE) and systemic chemotherapeutic agents (Cheng et al., 2020). However, prolonged use of chemotherapeutic drugs inevitably leads to HCC treatment resistance. Consequently, novel strategies to counteract the resistance of liver cancer to chemotherapeutic drugs are urgently required (Wang et al., 2023).

Extracellular vesicles (EVs) derived from cancer-associated fibroblasts (CAFs) transferred miR-1228-3p to HCC cells, promoting HCC proliferation, migration, and invasion by activating the PLAC8-mediated PI3K/AKT pathway, thereby increasing the resistance of patients to sorafenib (Zhang Y. et al., 2023). Yu Z *et al.* determined that miR-375, carried by EVs from bone marrow mesenchymal stem cells (MSCs), hinders HCC progression through the HOXB3-mediated Wnt/β-Catenin pathway (Yu et al., 2022). Li Y. H. et al. (2021). reported that miR-338-3p, secreted by bone marrow MSCs-derived EVs, inhibits HCC proliferation, invasion, and migration by targeting ETS1 and inducing cell apoptosis. Xu Y *et al.* isolated miR-451a-enriched EVs from human MSCs and co-cultured them with Hep3B and SMMC-7721 cell lines. Their findings revealed that EVs containing miR-451a significantly suppressed ADAM10 expression and epithelial-mesenchymal transition (EMT) while reversing paclitaxel resistance and promoting apoptosis in HCC cells (Xu et al., 2021). Zhou Y. et al. (2022). observed that CircZFR was highly expressed in cisplatin-resistant HCC cells. Meanwhile, the elevated expression of CircZFR in EVs originating from CAFs impedes the STAT3/NF-κB pathway, thereby enhancing cisplatin resistance in HCC cells. Liu C and colleagues discovered that CircTTLL5 was abundantly present in HCC tissues and cell-derived EVs. By inhibiting CircTTLL5 expression in EVs, HCC cell proliferation, *in vitro* metastasis, and tumor growth in nude mice were suppressed through the miR-136-5p/KIAA1522 signaling pathway (Liu et al., 2023). Yuan P *et al.* identified that circ_002136 found in HCC-derived EVs stimulates HCC progression through the miR-19a-3p/RAB1A pathway (Yuan et al., 2022). Fu X *et al.* found that EVs secreted by HCC cells downregulated SIK1 expression and enhanced the Wnt/β-catenin pathway by transporting miR-25, subsequently promoting HCC progression (Fu et al., 2022). You LN *et al.* found that the LINC00161, carried by EVs derived from HCC cells, activates the ROCK2 signal by inhibiting miR-590-3p, leading to increased proliferation, migration, and angiogenesis of HCC cells (You et al., 2021). Liu C *et al.* found that EVs carrying miR-30a-3p and deriving from HCC cells, reduced the migration, invasion, and metastasis ability of HCC cells by directly targeting SNAP23 (Liu et al., 2021). Li J *et al.* found that miR-15b from EVs secreted by As-THP-1 cells when transferred to HCC cells, promotes their proliferation, migration, and invasion through the Hippo pathway (Li J. et al., 2021). According to Huang M *et al.*, circGSE1 from EVs secreted by HCC promotes the progression of HCC by inducing Tregs enrichment and inhibiting anti-tumor immune responses via the miR-324-5p/TGFBR1/Smad3 pathway (Huang et al., 2022).

Zong QH *et al.* discovered that miR-452-5p, present in EVs derived from HCC, enhances the migration, invasion, and metastasis of HCC cells by targeting TIMP3 to induce M2 macrophage polarization (Zongqiang et al., 2022). Zhou J *et al.* found that EVs overexpressing PART1 promote M2 macrophage polarization as well as proliferation, migration, invasion, and EMT of HCC via the miR-372-3p/TLR4 pathway (Zhou J. et al., 2022). Zhang L *et al.* also observed that EVs from RBPJ over-expressing macrophages inhibit HCC progression through the hsa_circ_0004658/miR-499b-5p/JAM3 pathway (Zhang L. et al., 2022). Wang LP *et al.* identified that DLX6-AS1 secreted by EVs from HCC cells induces M2 macrophage polarization, which in turn promotes the migration and invasion of hepatocellular carcinoma through the miR-15a-5p/CXCL17 signaling pathway (Wang L. P. et al., 2021). Tian B *et al.* found that M2 macrophage-derived EVs carrying miR-660-5p also promote HCC progression by regulating KLF3 expression (Tian et al., 2021). Li W *et al.* found that EVs secreted by M2 macrophages elevate the stem cell characteristics of HCC through the miR-27a-3p/TXNIP pathway, thereby enhancing HCC malignancy (Li W. et al., 2021). In addition, Pu J *et al.* found that EVs derived from M2 macrophages promote CD8^+^T cell depletion in HCC through the miR-21-5p/YOD1/YAP/β-catenin pathway, contributing to HCC immune evasion (Pu et al., 2021).

Hepatic stellate cells (HSCs) serve as the primary source of CAFs in the liver, promoting liver fibrogenesis through extracellular matrix remodeling (Higashi et al., 2017; Wu et al., 2021). Zhang X *et al.* discovered through *in vitro* co-culture of HCC cells and HSCs, that miR-148a-3p in EVs derived from HSCs impedes HCC malignancy through the PI3K/Akt/ITGA5 signaling pathway (Zhang X. et al., 2022). Moreover, Liu L *et al.* determined that circWDR25 in EVs secreted by HSCs stimulates the proliferation and invasion of HCC cells through miR-4474-3p/ALOX15 and EMT pathway while significantly increasing PD-L1 and CTLA-4 expression in HCC and HSC cells to promote immune escape (Liu et al., 2022). Xia Y *et al.* found that SMO can be transferred from HCC cells to HSCs via EVs. This transfer further elevates SMO expression and stimulates HSC activation by activating the Hedgehog pathway, which in turn regulates the GLI1/MIRLET7BHG/miR-330-5p/SMO pathway. Activated HSCs then stimulate HCC malignancy (Xia et al., 2021).

These findings suggest that non-coding RNAs in EVs not only modulate HCC malignancy and chemotherapeutic sensitivity through related pathways but also mediate the biological behavior of HCC by regulating macrophage polarization and affecting HSC activity. These non-coding RNAs which were loaded in EVs and can regulate the malignant progress of HCC were also summarized in Table 1.

**TABLE 1:** The non-coding RNA loaded in EVs can regulate the malignant progress of HCC.

Name	Origin	Biological function	Regulation mechanism	Reference
miR-1228-3p	EVs derived from cancer-associated fibroblasts	Promoting HCC proliferation, migration, and invasion	Activating the PLAC8-mediated PI3K/AKT pathway	Zhang J. et al. (2023)
miR-375	EVs from bone marrow mesenchymal stem cells	Hindering HCC progression	Activating the HOXB3-mediated Wnt/β-Catenin pathway	Yu et al. (2022)
miR-338-3p	EVs from bone marrow mesenchymal stem cells	Inhibiting HCC proliferation, invasion, migration, and inducing apoptosis	Targeting ETS1	Li J. et al. (2021)
miR-451a	EVs from bone marrow mesenchymal stem cells	Reversing paclitaxel resistance and promoting apoptosis in HCC cells	Suppressing ADAM10 expression and epithelial-mesenchymal transition	Xu et al. (2021)
CircZFR	EVs derived from cancer-associated fibroblasts	Enhancing cisplatin resistance in HCC cells	Impeding the STAT3/NF-κB pathway	Zhou J. et al. (2022)
CircTTLL5	EVs derived from HCC cells	Promoting HCC cell proliferation, *in vitro* metastasis, and tumor growth in nude mice	miR-136-5p/KIAA1522 signaling pathway	Liu et al. (2023)
Circ_002136	EVs derived from HCC cells	Stimulating HCC progression	miR-19a-3p/RAB1A pathway	Yuan et al. (2022)
miR-25	EVs derived from HCC cells	Promoting HCC progression	Downregulating SIK1 expression and enhancing the Wnt/β-catenin pathway	Fu et al. (2022)
LINC00161	EVs derived from HCC cells	Leading to increased proliferation, migration, and angiogenesis of HCC cells	Activating the ROCK2 signal by inhibiting miR-590-3p	You et al. (2021)
miR-30a-3p	EVs derived from HCC cells	Reducing the migration, invasion, and metastasis ability of HCC cells	Targeting SNAP23	Liu et al. (2021)
miR-15b	EVs secreted by As-THP-1 cells	Promoting HCC cells proliferation, migration, and invasion	Through the Hippo pathway	Li W. et al. (2021)
circGSE1	EVs derived from HCC cells	Promoting the progression of HCC	Inducing Tregs enrichment and inhibiting anti-tumor immune responses via the miR-324-5p/TGFBR1/Smad3 pathway	Huang et al. (2022)
miR-452-5p	EVs derived from HCC cells	Enhancing the migration, invasion, and metastasis of HCC cells	Targeting TIMP3 to induce M2 macrophage polarization	Zongqiang et al. (2022)
miR-660-5p	EVs derived from M2 macrophage	Promoting HCC progression	Regulating KLF3 expression	Tian et al. (2021)
miR-148a-3p	EVs derived from hepatic stellate cells	Impeding HCC malignancy	Through the PI3K/Akt/ITGA5 signaling pathway	Zhang X. et al. (2022)
circWDR25	EVs derived from hepatic stellate cells	Stimulating the proliferation and invasion of HCC cells	Through activating miR-4474-3p/ALOX15 and epithelial-mesenchymal transition pathway	Liu et al. (2022)

## 3 EVs can serve as biomarkers for HCC diagnosis

HCC is the most prevalent type of primary liver cancer. Due to the limited sensitivity and specificity of serum α- Fetal protein (AFP) diagnostic methods, further screening of HCC diagnostic markers with enhanced accuracy is still required. Numerous studies have confirmed that EVs can serve as biomarkers for HCC diagnosis and demonstrate great potential for practical applications. Sun N *et al.* devised a scoring system based on membrane proteins found in HCC-secreted EVs, which proved effective in diagnosing early HCC (Sun et al., 2023). In addition, abnormal membrane protein glycosylation has been proven to be a marker for diagnosing malignant tumors. Li D *et al.* conducted a comprehensive analysis and screening of urinary EVs focusing on N-glycosylation levels. Their results showed that the expression of glycoproteins LG3BP, PIGR, and KNG1 were significantly upregulated in these EVs derived from the urine of patients with HCC, while the expression of ASPP2 was significantly downregulated in these EVs.

Furthermore, it was shown that the abnormal glycosylation of EV membrane proteins holds potential as an effective noninvasive biomarker for HCC diagnosis (Li et al., 2023). Lin J *et al.* isolated EVs from HCC and adjacent liver tissues for miRNA-level sequencing. miRNA levels were also sequenced using EVs isolated from the serum of HCC patients and healthy volunteers. Analysis of sequencing results and experimental validation revealed that hsa-miR-483-5p was the only differentially expressed miRNA detected in EVs from both HCC tissue and the plasma of HCC patients. Further research found that miR-483-5p was highly expressed in EVs of HCC and promoted HCC cell proliferation by binding to CDK15 and downregulating CDK15 expression, ultimately mediating the malignant progression of HCC. This study demonstrated that hsa-miR-483-5p is a potential biomarker for the diagnosis of HCC (Lin et al., 2022). In addition, Yokota Y *et al.* found that miR-638 in EVs secreted from highly metastatic HCC cells increased vascular permeability by downregulating the expression of VE cadherin and ZO-1 in vascular endothelial cells and then promoted HCC cell metastasis to affect the progression of HCC.

Simultaneously, miR-638 in EVs has been shown to serve as an independent prognostic factor for HCC patients and a biomarker for HCC diagnosis (Yokota et al., 2021). The above results indicated that EVs hold potential as efficient biomarkers for HCC diagnosis. However, further research is necessary to screen and identify more effective and noninvasive EV-related biomarkers for early diagnosis of HCC.

## 4 Novel methods for HCC nanotherapy based on EVs

Numerous studies have explored new EV-based methods of HCC nanotherapy. Zhang J *et al.* constructed an extracellular delivery platform using EVs derived from HEK293F. They improved the pharmacokinetic curve of IL-12 targeting HCC by optimizing the surface integrins and N-glycans of EVs. The resulting EVs exhibited better bioavailability and enhanced the cyclic half-life of IL-12. Furthermore, single peptide antibodies targeting GPC3 significantly improved the targeting efficiency and accuracy of EVs in HCC cells loaded with IL-12, achieving more significant anti-tumor effects (Zhang J. et al., 2023). Wang C *et al.* loaded the Norcantharidin (NCTD) anti-tumor drug into EVs derived from bone marrow MSCs. They found that the BMSC-Exo-NCTD delivery system effectively promoted the absorption of NCTD by HCC HepG2 cells, induced HepG2 cell cycle arrest, reduced HepG2 proliferation, and increased HepG2 apoptosis. Moreover, compared to treatment with NCTD alone, BMSC-Exo-NCTD exhibited better anti-tumor effects (Liang et al., 2021). In addition, *in vivo* detection results using probe Cy5.5 showed that the BMSC-Exos vector had an *in situ* homing effect on mouse HCC and did not exhibit physical toxicity (Liang et al., 2021). Thapa N *et al.* loaded miR-335, which exerted the anticancer effect of HCC, into EVs to prevent its degradation and promote its effective delivery to the target site, thereby inhibiting the growth of HCC cells (Thapa et al., 2023). Chen *et al.* loaded asiatic acid into EVs derived from HCC and found through *in vitro* experiments that EVs loaded with asiatic acid exerted significant anti-HCC effects by regulating the TGF signaling pathway to inhibit the EMT process. Zhou X *et al.* isolated EVs derived from HCC and then fused EVs membranes with phospholipids to create new EVs membrane mixed lipid nanocapsules, achieving precise tumor targeting and efficient siRNA transfection. Compared with liposomes, EV membrane containing siRNA mixed with lipid nanocapsules increased transfection efficiency by 1.7 times. In addition, effective oncogene silencing at the tumor site further enhances its anti-HCC effect (Zhou X. et al., 2022). Deng J *et al.* found that CD38 was highly expressed in HCC tissues and cell lines. Lowering CD38 levels promotes macrophage phagocytosis by inhibiting adenosine, which leads to the inhibition of growth and metastasis in HCC. Deng J *et al.* used EVs derived from MSCs as carriers for siCD38, enabling its efficient and precise delivery to HCC cells. The results showed that EVs/siCD38 inhibited HCC progression and reversed the therapeutic resistance to PD-1/PD-L1 inhibitors (Deng and Ke, 2023). Yang C *et al.* loaded Doxorubicin into purified MSC-EVs and improved encapsulation and drug loading efficiency through ultrasound technology, forming a delivery system E-Dox. After removing sialic acid from MSC-EVs by neuraminidase, the receptor cells showed increased endocytosis of E-Dox. They effectively transported Dox to HCC cells, inhibiting their proliferation and migration and promoting their apoptosis. The safety of E-Dox in the treatment of HCC has been verified using a mouse model (Yang et al., 2022). CRISPR-Cas9 genome editing has emerged as a powerful therapeutic tool. However, its clinical application has been limited by the lack of a safe and efficient *in vivo* delivery system, particularly for tissue-specific vectors. Cas9 ribonucleoprotein (RNP) was loaded into EVs isolated and purified from HSC. The resulting EVs-RNP significantly enhanced the accumulation of RNP in HCC cells.

**FIGURE 1:**
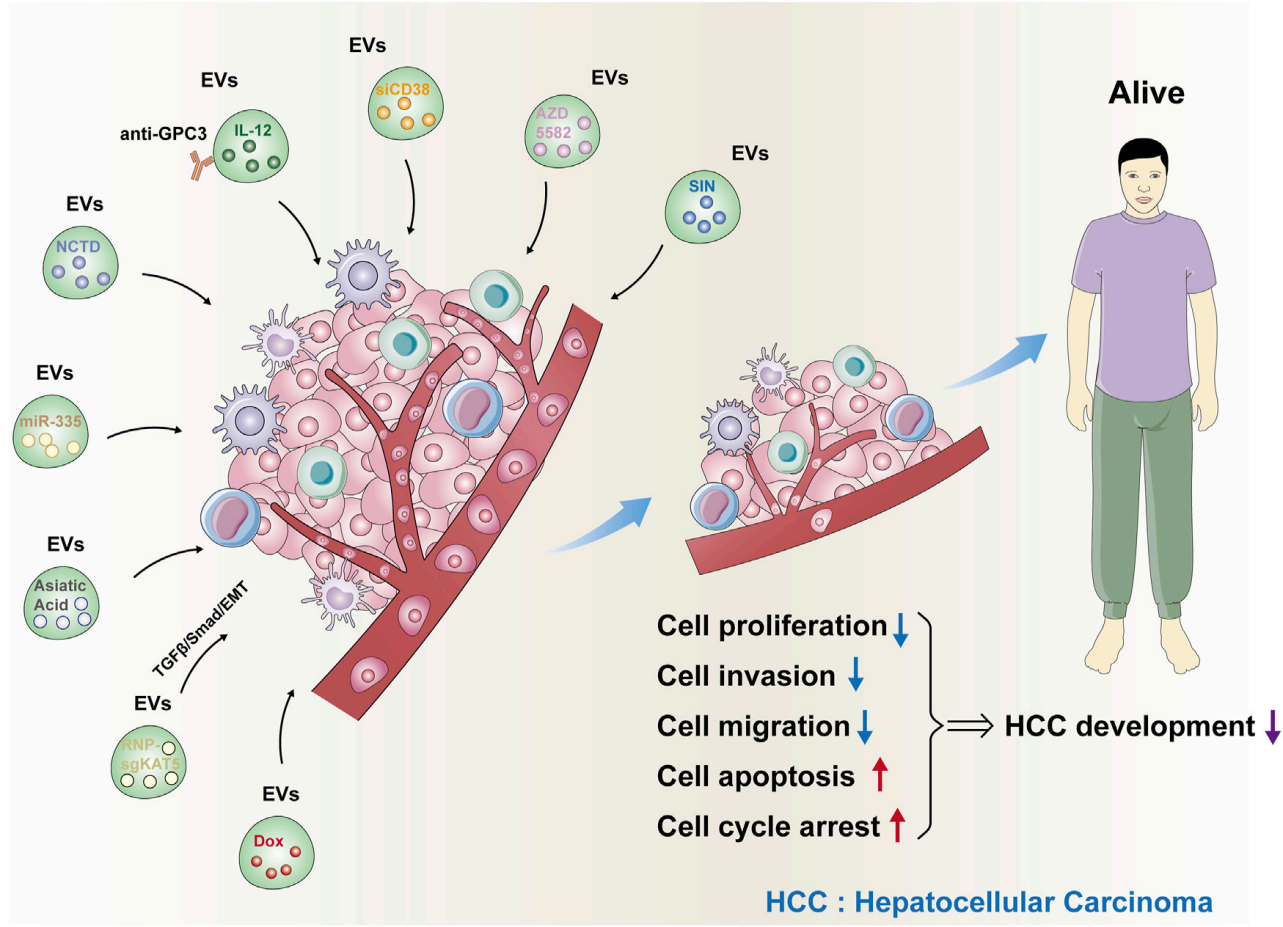
New methods for HCC nanotherapy based on EVs. The nine kinds of new methods for HCC nanotherapy based on EVs were summarized. These EVs loaded with responding drugs could significantly inhibit cell proliferation, invasion, and migration and induce cell apoptosis and cell cycle arrest. As a result, HCC development was significantly suppressed.

Subsequently, Wan T *et al.* designed sgRNA to target the lysine acetyltransferase 5 (KAT5) required for HCC growth. After loading sgKAT5 into EVs-RNP, the resulting EVs-RNP- sgKAT5 efficiently accumulated in HCC cells. By targeting KAT5 and downregulating its expression, the EVs-RNP- sgKAT5 was able to inhibit HCC growth (Kwan et al., 2020; Wan et al., 2022). Su K *et al.* found that compared to the potent antagonist AZD5582 using apoptotic protein inhibitors alone, loading AZD5582 into EVs significantly enhanced its anti-HCC effect and overcame TRAIL resistance without any significant adverse reactions (Su et al., 2022). Sinomenine (SIN) demonstrated significant anti-HCC activity *in vitro*. However, its clinical efficacy is limited due to its low bioavailability. Wang Y *et al.* prepared EVs (Exo-SIN) loaded with SIN. Exo-SIN could release SIN in a continuous and slow manner in an experimental model simulating body fluid and the tumor microenvironment. Moreover, compared to the solo use of SIN, Exo-SIN significantly inhibited the proliferation and migration of HepG2 cells, caused cell cycle arrest, and induced apoptosis. Furthermore, after treatment with Exo-SIN, the expression of survivin (a key protein for HCC cell survival) decreased significantly. The above results indicated that Exo-SIN based on EV nanocarriers improved the bioavailability of SIN in the treatment of HCC and provided an effective treatment platform for HCC (Wang Y. et al., 2021). As for other advantages of these new methods for HCC nanotherapy based on EVs, they may be more effective and safer compared with traditional therapeutic methods. Also, they huged the ability of targeting capabilities, low toxicity and modifiability. Nanotherapy based on EVs may even regulate angiogenesis, immune response, and tumor metastasis to control the development of HCC and significantly improve the survival outcomes of patients with HCC. We believe that these new methods for HCC nanotherapy based on EVs have excellent application prospects. These new methods for HCC nanotherapy based on EVs were also summarized and presented in Figure 1.

## 5 Summary and outlook

This article focuses on the important role of the non-coding RNA carried by EVs in HCC progression, the potential of EVs as biomarkers for HCC diagnosis, and provides an overview of the current EV-based nanotherapy for HCC, hoping to provide a reference for clinical diagnosis and treatment of HCC. However, the current EV-based HCC nanotherapy methods are limited in certain aspects. Similar to most conventional therapeutic nanoparticles, even with EV-based drug delivery systems, challenges such as rapid half-life liver clearance after intravenous injection and limited bioavailability persist. Consequently, future research should focus on developing novel nanotherapy methods based on EVs with longer half-lives, slower liver clearance rates, and higher bioavailability. Ultimately, this will pave the way for new clinical treatment options for HCC, significantly improving its efficacy.
